# Microbiological Response to Periodontal Therapy: A Retrospective Study

**DOI:** 10.2174/1874210601812010837

**Published:** 2018-10-25

**Authors:** Vittorio Checchi, Gaia Pascolo

**Affiliations:** 1 Department of Restorative Dentistry, University of Bologna, DIBINEM, Bologna, Italy; 2 Department of Periodontology and Implantology, University of Bologna, DIBINEM, Bologna, Italy

**Keywords:** Microbiology, Periodontal therapy, Periodontal pathogens, Retrospective study, Microbiological test, Periodontal surgery

## Abstract

**Background:**

Periodontitis is a multifactorial infection caused by a complex of pathogenic bacterial species that induce the destruction of periodontal structures.

**Objective:**

The aim of this study is to evaluate the presence and bacterial load of six periodontal pathogens bacteria, measured at initial visit and after osseous surgery in patients affected by chronic periodontitis and treated between 2005 and 2007.

**Methods:**

This cohort study was carried out on a sample of 38 consecutive patients affected by severe chronic periodontitis, diagnosed at baseline on the basis of probing depths equal to 6.68 ± 1.47 mm. On each subject, a microbiological test was performed before periodontal initial therapy and after osseous surgery (one year later). Five compromised teeth were chosen for each patient (the same teeth, before and after surgery), for a total of 190 teeth. Real-time PCR based analysis computed total bacterial load of the samples and quantified six periodontal pathogens: *Actinobacillus actinomycetemcomitans, Porphyromonas gingivalis, Tannerella forsythia*, *Treponema denticola, Fusobacterium nucleatum* and *Prevotella intermedia*. Data collection was made consulting medical charts.

**Results:**

Pocket probing depth reduction after surgery was 4.50 ± 1.54 mm (*p*=0.0001). The mean number of sites with bleeding at baseline was 2.08 ± 1.17 and 0.58 ± 1.00 after surgery (*p*=0.001). The mean number of sites with suppuration at baseline was 0.26 ± 0.86 and 0 after surgery (*p*=0.02). Cell count of each pathogen and total cell count were significantly higher at baseline than after surgery. Almost all bacteria presented a mean percentage reduction equal to that of the total count, except for *Aa* and *Pi,* which seemed to show a greater resistance. The difference of bacterial load, both before and after surgery, between smokers and non-smokers was not statistically significant (*p*<0.05). A statistically significant correlation was detected between pocket probing depth variation and bleeding on probing variation before and after the surgery, controlling for age (r=0.6, *p*=0.001). No significant correlations were observed between pocket probing depth and bacterial loads, except for *Pg* (r=0.5, *p*=0.001), *Tf* (r=0.6, *p*=0.001) and *Td* (r=0.4, *p*=0.02).

**Conclusions:**

Reduction of presence and bacterial load of the examined periodontal pathogens bacteria after osseous surgery, along with periodontal pocket reduction, appeared to be essential to achieve and maintain periodontal stability over years.

## 1 INTRODUCTION

Periodontitis is a multifactorial infection caused by a complex of pathogenic bacterial species that induce the destruction of periodontal structures, including tooth-supporting tissues, alveolar bone and periodontal ligament [1]. Some co-factors such as smoking, genetic susceptibility and host response, can increase and exacerbate microbial actions [2, 3]. Periodontal diseases may arise as gingivitis or periodontitis: The first can heal with the *restitutio ad integrum* of all tooth-supporting tissues, the second instead always develops an irreversible lesion [4]. Periodontitis is site-specific: each tooth and its surfaces may be involved with different severity. Consequently, an accurate diagnosis is needed for each case and each tooth.

The current definitions of periodontitis were introduced at the 1999 World Workshop for the Classification of Periodontal Diseases and Conditions [5]. Chronic periodontitis is a progressive disease that alternates silent and acute phases; aggressive periodontitis instead, is a highly destructive form of periodontitis. Both can be localized or generalized (respectively ≤ 30% of sites involved or > 30% of sites involved). Severity can be classified on the basis of the amount of Clinical Attachment Loss (CAL loss) in slight (1 or 2 mm CAL loss), moderate (3 or 4 mm CAL loss) or severe (≥ 5mm CAL loss) [6].

In the oral cavity we can find almost 700 bacterial species: microorganisms show a structural organization in the biofilm where they compete, coexist and⁄or synergize, leading to this chronic disease [7-9]. This well-structured microbiological biofilm is able to colonize the sulcular regions between the tooth surface and the gingival margin [10]: the first species to reach these areas are gram-positive cocci and rods, followed by gram-negative cocci and rods, then fusobacteria, filaments, and finally spirillae and spirochetes [11].

The use of checkerboard DNA-DNA hybridization method made it possible to identify new bacterial species [12] that Socransky *et al*., clustered in complexes characterized by different virulence factors: Actinomyces species, Purple complex, Yellow complex, Green complex, Orange complex and Red complex [13].

Since bacterial microflora was found to differ between active and inactive sites, microbiological monitoring plays an important role not only for diagnosis but also for the choice of periodontal therapy [14, 15]. During initial periodontal therapy, patients are usually treated with Scaling and Root Planing (SRP) to remove supra- and sub-gingival plaque and calculus, etiological agents of periodontal disease [16], and to obtain an increase of clinical attachment level. In case of persisting deep (> 4 mm) pockets after initial therapy, the sites need to be treated with resective osseous surgery [17].

The aim of this study is to evaluate the presence and bacterial load of six periodontal pathogenic bacteria detected at initial visit and after osseous surgery, in patients previously treated for chronic periodontitis. The null hypothesis was that no difference is observed between baseline and first visit in the presence and bacterial load of six pathogenic bacteria.

## 2 MATERIALS AND METHODS

This cohort study was carried out on a sample of 38 consecutive patients affected by chronic periodontitis, treated from 2005 to 2007 and attending a periodontal private practice (V.C.) in Bologna, Italy. Data collection was made in November 2013, consulting medical charts. Subjects were enrolled according to the following inclusion criteria:

- clinical diagnosis of chronic periodontal disease [18],
- presence at least of 12 teeth (excluded third molars)
- no periodontal treatment in the previous six months,
- no systemic diseases (as diabetes, arthritis, ulcerative colitis, Crohn disease, HIV, cancer, cardiac pathologies),
- no antibiotic therapy in the previous 4 weeks,
- no ongoing pregnancy,
- presence, in the medical records, of microbiological tests performed before and after osseous surgery.

All selected subjects signed an informed consent form. This study was approved by Bologna-Imola Ethical Board Committee (Prot. N. 956/CE - 02/11/2016).

At the initial visit, each patient had been clinically and radiographically examined and Probing Pocket Depth (PPD), Bleeding On Probing (BOP) and suppuration data were recorded. Each clinical parameter was evaluated with a periodontal standard calibrate probe (Hu Friedy PCP11) that measured the depth from the gingival margin to the deepest point of the pocket. Moreover, patient’s age, gender and smoking habits were recorded.

Microbiological test was performed during the first appointment, before oral hygiene instructions and supra-gingival gross scaling. SRP was carried out in 4 successive sessions, once a week, one quadrant per week. Finally, after periodontal re-evaluation, patient underwent osseous surgery in the case of the presence of pocket sites with PPD > 4 mm and BOP +. One year after surgical treatment, a second microbiological test was performed.

On each subject participating in the study, a microbiological test had been performed before periodontal initial therapy and after osseous surgery (about one year from baseline).

For the microbiological test, five compromised teeth had been chosen for each patient (the same teeth, before and after surgery), at least one tooth for each quadrant, for a total count of 190 teeth.

Supra-gingival plaque and/or calculus were removed with Gracey’s curette. After that, the site was isolated from saliva by some cotton rolls and then delicately dried. For each site, a sterile paper cone was inserted in the sulcus at pocket depth and removed after 10 seconds. All 5 paper cones were collected, then placed in a unique sterile tube and sent to the laboratory (GmbH International GABA, Munster, Germany) for further microbiological evaluations. Samples were reduced with Wilkins-Chalgren-suspension and vortexed for 30 seconds to obtain a homogeneous suspension: 0.5 ml of the suspension was used for real-time PCR analysis. The cells were then centrifuged (15.000 turns at 41°C) for ten minutes and later subjected to automatic process analysis (Meridol Analysis^®^ Perio Diagnostics GABA International, Switzerland). Real-time PCR based analysis [19] was developed and validated (Carpegen GmbH). This method computes total bacterial load of the sample and quantifies six periodontal pathogens with a sensibility of 100 cells per type of pathogens: *Actinobacillus actinomycetemcomitans (Aa)*, *Porphyromonas gingivalis (Pg)*, *Tannerella forsythia (Tf)*, *Treponema denticola (Td)*, *Fusobacterium nucleatum (Fn)* and *Prevotella intermedia (Pi)*.

### 2.1 Statistical Analysis

The sample size was determined by hypothesizing a mean difference between baseline and 1-year measurements of 0.5 (Cohen’s medium effect size) [20] with a standard deviation of 1. A sample size of 38 subjects was obtained with a power of 85% at an α-level of 0.05. In each subject, 5 sites were examined: median value of probing depth, number of sites with bleeding on probing and number of sites with suppuration were computed for each patient and used for statistical evaluation. Mean ± standard deviation was used to describe the data. The comparison between bacterial load at baseline and after one year was performed by using Wilcoxon test for paired data, after having ascertained the non-normality of the distributions of the bacterial loads by means of Shapiro-Wilks test (*p*=0.0001). Following the Socransky principles, the studied organisms were grouped into red complex (*Pg, Td, Tf*) and orange complex (*Pi, Fn*). Furthermore, the presence of *Actinomyces* (*Aa*) [13] was also determined.

The percentage decrement of the bacterial load was computed with the formula: [(bacterial load after 1 year - bacterial load at baseline)*100/ bacterial load at baseline]. Kruskal-Wallis non-parametric analysis of variance was used for the comparisons between the percentage mean decrements of the bacterial loads among the six periodontal pathogens. McNemar chi square test was carried out aiming to compare the frequency of detection of the six periodontal pathogens before and after surgery. Exact binomial 95% confidence intervals of the proportions were also computed. Partial correlation coefficient was used to measure the association between variation of probing pocket depth and the number of sites with bleeding or suppuration, controlling for the age of the patients. Alpha level was *a priori* set at 0.05.

## 3 RESULTS

Thirty-eight patients (15 males and 23 females) were examined, 16% were smokers and 84% non-smokers, and the mean age was 47 ± 13 years.

Patients were diagnosed with severe chronic periodontitis at baseline on the basis of probing depth equal to 6.68 ± 1.47 mm. The reduction of pocket probing depth after surgery was 4.50 ± 1.54 mm and it was statistically significant (*p*=0.0001). Thirty-eight % of patients presented a number of sites with BOP greater or equal to 3; the mean number of sites with bleeding at baseline was 2.08 ± 1.17 and 0.58 ± 1.00 after surgery, with a statistically significant decrease (*p*=0.001). Sixteen % of patients presented a number of sites with suppuration greater or equal to 1; the mean number of sites with suppuration at baseline was 0.26 ± 0.86 and 0 after surgery, with a statistically significant decrease (*p*=0.02).

Bacterial loads at baseline and after 12 months are compared in Table **1**. Cell count of each pathogen and total cell count were significantly higher at baseline than after surgery; the highest load was presented by *Pg* and the lowest by *Aa*. Grouping the pathogens, it emerged that even red and orange complex bacterial load significantly decreased from baseline to the first control.

The highest mean percentage of decrease from baseline to one year control was presented by *Fn,* whereas the lowest was presented by *Aa*; statistically significant differences of percentage mean decrements were observed among the six periodontal pathogens (*p*=0.004) as shown in Table **2**. Almost all bacteria presented a mean percentage reduction equal to that of the total count, except for *Aa* and *Pi,* which seemed to show a greater resistance.

The frequency of detection of the six bacteria is presented in Table **3**. The amplitude of confidence intervals suggests that these estimates are not precise, even if it shows, at baseline, the highest prevalence of the red complex and the highest resistance of *Fn*. However, the frequencies of detection significantly decreased from baseline to one year after surgery, except for *Aa* and *Fn*.

The difference of bacterial load, both before and after surgery, between smokers and non-smokers was not statistically significant (*p*>0.05).

Furthermore, a statistically significant correlation was detected between pocket probing depth variation and bleeding on probing variation before and after the surgery, controlling for age (r=0.6, *p*=0.001). No significant correlations were observed between pocket probing depth and bacterial loads, except for *Pg* (r=0.5, *p*=0.001), *Tf* (r=0.6, *p*=0.001) and *Td* (r=0.4, *p*=0.02).

## 4 DISCUSSION

The purpose of this retrospective cohort study was to compare data on the frequency of detection and bacterial cell count of six important periodontal pathogens at the initial visit and after osseous surgery, in patients affected by chronic periodontitis.

Our results showed that the cell count of each pathogen and the total cell count were significantly higher at baseline than at the first control, after 1 year. The highest loads were presented by *Pg, and* the lowest by *Aa*. By grouping the pathogens, it emerged that both red and orange complex bacterial loads significantly decreased from baseline to the first control. The red complex, composed of *Pg, Td* and *Tf* and the orange complex, composed of *Pi* and *Fn*, were demonstrated to be strongly associated with periodontal disease [13].

Levy *et al*., showed that 12 months after surgery, not only the mean total DNA probe count was significantly reduced (*p*<0.01), but also each single bacteria species diminished significantly: *Pg* (*p*<0,05), *Td* (*p*<0,01), *Fn* (*p*<0.001) and *P. nigrescens* (*p*<0,01) [21].

These findings also confirm the data from Mombelli *et al.*, who found reductions in levels, proportions and prevalence of gram-negative species of the red and orange complexes after surgery. In this study, after 12 months, *Pg* was reduced from 40% to 4% and *Fusobacterium sp.* was reduced from 80% to 42%. Considering the mean values, the authors reported that *Pi*, *Fusobacterium sp.* and *Campylobacter rectus* had a significant decrease, respectively from 1.8 x 10^6^ to 1.9 x 10^5^, from 1.1 x 10^6^ to 5.5 x 10^5^ and from 8.9 x 10^5^ to 1.0 x 10^5^ [22].

Other authors reported that after periodontal osseous surgery, *Aa* and *Pg* were not detected in any patient 6 months after the surgery, respectively from 1% to 0% and from 5% to 0%. In addition, *Pi* and *Fusobacterium sp*. were recovered in a low proportion of patients after surgery [23].

Kyriazis *et al*., found that there was a constant decrease in red complex periodontal pathogens after periodontal osseous surgery: *Pg* was reduced from 3.1 x 10^5^ to 1.7 x 10^5^, *Tf* was reduced from 6.7 x 10^5^ to 4.2 x 10^5^, while *Td* remained almost stable (3.43 x 10^5^ to 3.95 x 10^5^) [24].

To explain significant reduction of red and orange complexes, Horibe explained that tissues management during surgical procedures may lead to an altered host immunologic response to pathogenic species, which later on may exhibit beneficial clinical effects even in sites that were not receiving periodontal surgery [25].

In our study, the highest mean decrease from baseline to after surgery was presented by *Fn*, with a percentage of 79%, whereas the lowest by *Aa*, with a percentage of 23%. These data are in disagreement with the results by Levy *et al*., where the proportional reductions begun to reverse from 9 to 12 months for *Fn* (increase of 31%) and *P. nigrescens* (increase of 59%*)* [20]. However, after using the checkerboard DNA–DNA hybridization technique, the authors confirmed that surgery led to a further reduction of periodontal pathogens compared to SRP alone.

Various studies investigated the beneficial effect in bacterial microflora synthesis derived from apically positioned flap, associated with osseous surgery [21, 22]. Moreover, Danser *et al*., reported that modified Widman flap surgery leads to a reduction of the prevalence of periodontal pathogens [26]. The authors examined the prevalence of *Aa, Pg* and *Pi* 3 months after periodontal surgery: the prevalence of *Aa* in the sub-gingival plaque was significantly reduced with a mean percentage of 1.2% (*p* = 0.02). A further significant reduction, with a mean percentage of 0.2% (*p*=0.003), was seen in the prevalence of *Pg* after periodontal surgery. Additionally, the prevalence of *Pi* was reduced with a mean percentage of 0.2% (*p* < 0.01) [25].

It has also been suggested that environmental changes, consequences of periodontal surgery, may lead to a shift in the sub-gingival microflora and that the final bacterial composition is more compatible with an oral health status [27].

Concerning clinical parameters and in agreement with other data [20], in our study there was a significant decrease in mean probing depth in surgically treated sites: from 6.68 ± 1.47 mm at baseline to 4.5 ± 1.54 mm after 1 year. In a similar study, Tuan et al showed a mean pocket depth of 5.5 mm at baseline, of 1.9 mm after 1 month, of 2.0 mm after 3 months and of 2.1 mm after 6 months, in sites treated with osseous surgery [22]. In the study of Danser *et al*., the probing depth decreased from 7.0 mm at baseline to 3.9 mm after surgery. Precisely, the mean reduction of pocket probing depth after SRP was 1.4 mm, and after periodontal surgery, probing depth decreased for an additional 1.3 mm [25]. Another research group reported that from baseline to post initial therapy, there had been a rapid pocket depth decrease (from 3.2 mm to 2.4 mm); on the contrary, from post initial therapy to the post-surgical examination, there had been no statistically significant decrease (from 2.4 mm to 2.2 mm) [28]. The authors obtained a greater mean reduction after SRP because tissue inflammation before initial preparation was greater than that observed before surgical therapy.

In our study we found a statistically significant reduction (*p*=0.001) of sites with BOP: precisely, the mean decrease after surgery was 0.58 ± 1.00. These results are in agreement with others where the mean BOP was 1.8 at baseline and 1.1 after surgery (*p*<0,0001) [20, 25]. Also Tuan *et al*., reported that BOP decreased from 30.4 ± 12.8 to 6.3 ± 9.4 (*p*=0,05) after 6 months from surgery [22].

In few studies, Tanner *et al*., showed that in bleeding sites, there was massive presence of gram-negative bacteria including *F. nucleatum* species, *T. forsythia*, *Pi, P. nigrescens* and *Pg* [29, 30]. In addition, other authors reported that three bacteria of red complex species (*T. forsythia, Pg, Td)* and different bacteria of the orange complex were interconnected to the presence of bleeding on probing [11, 31]. Even though the presence of BOP is not a reliable predictor or indicator for the loss of additional periodontal attachment [32], it has been verified that the presence of some specific periodontal pathogens (*e.g. T. forsythia*) may be connected with some sites that tend to convert to periodontitis [33].

Clinical studies have shown that there is a relationship between advanced age and increased pocket depth, and between advanced age and increased number of sites with BOP [34, 35]. In our study, we found that a reduction of pocket depth between the initial visit and after surgery corresponded to a reduction in the number of sites with BOP (r = 0.5). This correlation may indeed be influenced by the relationship that exists between the two clinical parameters that we analyzed and the age of the patients treated. Therefore, we used the bivariate correlation coefficient to make statistically constant age-variable nullifying its influence on the correlation. After this procedure, the correlation between pocket depth and BOP was found to be stronger (r=0.6), passing from 25% of cases to 36%.

One of the greater risk factors for periodontitis arises and progression is represented by cigarette smoking. Tobacco smoking alters the environment of the oral cavity, influencing the development of sub-gingival microbial pathogens (*Td* and *Pg*) that resulted significantly higher in smokers than in non-smokers [36, 37]. Renvert *et al*., showed that current cigarette smokers respond less well than non-smokers to periodontal therapy such as SRP and osseous periodontal surgery [38]. The observation of no statistically significant difference in the cell counts between smokers and non-smokers in our research may be due to the low number of smokers (16%) present.

## CONCLUSION

The evaluation of presence and bacterial load of all the examined periodontal pathogens bacteria before periodontal treatment and after osseous surgery showed a significant decrease after surgical therapy. These results, along with periodontal pocket reduction, seem to be essential to achieve and maintain periodontal stability over years. Further analyses on larger samples are needed to confirm the findings of this retrospective study.

## Figures and Tables

**Table 1:** Comparisons between the mean bacterial loads of the examined microorganisms at baseline and after 1 year (* Standard Deviation; ** Total cell count represents the whole bacterial load of the oral cavity)

**Microorganisms**	**Baseline** Mean (SD)*	**After 1 Year** Mean (SD)*	**Wilcoxon Test *p*=**
Actinobacillus actinomycetemcomitans (Aa)	3,5 x10^4^ (8,8 x 10^4^)	1,5x10^3^ (8,0 x 10^3^)	0,005
**Red Complex**	-		
Porphyromonas gingivalis (Pg)	7,2 x 10^6^ (1,3 x 10^7^)	1,5 x 10^6^ (7,5 x 10^6^)	0,001
Tannerella forsythensis (Tf)	3,0 x 10^6^ (5,3 x 10^6^)	8,3 x 10^5^ (3,3 x 10^6^)	0,001
Treponema denticola (Td)	4,9 x 10^6^ (1,2 x 10^7^)	3,5 x 10^5^ (1,3 x 10^6^)	0,001
Total red complex	1,5 x 10^7^ (2,6 x 10^7^)	2,7 x 10^6^ (1,2 x 10^7^)	0,001
**Orange Complex**	-		
Fusobacterium nucleatum (Fn)	1,1 x 10^6^ (2,3 x 10^6^)	1,7 x 10^5^ (3,8 x 10^5^)	0,012
Prevotella intermedia (Pi)	1,5 x 10^6^ (3,0 x 10^6^)	3,4 x 10^5^ (8,7 x 10^5^)	0,005
Total orange complex	2,6 x 10^6^ (4,3 x 10^6^)	5,1 x 10^5^ (9,2 x 10^5^)	0,002
Total cell count**	1,4 x 10^8^ (1,9 x 10^8^)	2,7 x 10^7^ (6,7 x 10^7^)	0,001

**Table 2:** Comparisons between the mean percentage decrements of bacterial loads of the examined microorganisms from baseline to 1 year (** Total cell count represents the whole bacterial load of the oral cavity)

**Microorganisms**	**Mean Decrement (%)**	**Standard Deviation**	**Kruskal-Wallis Non Parametric Analysis of Variance** ***p=***
Actinobacillus actinomycetemcomitans (Aa)	23,14	40,57	-
Porphyromonas gingivalis (Pg)	69,34	40,81	-
Tannerella forsythensis (Tf)	72,15	35,71	0,004
Treponema denticola (Td)	74,44	39,12	-
Fusobacterium nucleatum (Fn)	78,63	29,52	-
Prevotella intermedia (Pi)	53,37	48,26	-
Total cell count**	76,89	29,90	-

**Table 3:** Comparisons between the frequency of detection (%) of the examined microorganisms at baseline and after 1 year with their exact binomial 95% confidence interval (95% CI).

**Microorganisms**	**Baseline (%)** **(95% CI)**	**After 1 Year (%)** **(95% CI)**	**McNemar Test** ***p=***
*Actinobacillus actinomycetemcomitans (Aa)*	26 13-43	13 4-28	0.06
**Red Complex**	-		
*Porphyromonas gingivalis (Pg)*	82 66-92	40 24-57	0.001
*Tannerella forsythensis (Tf)*	90 75-97	53 36-69	0.001
*Treponema denticola (Td)*	82 66-92	42 26-59	0.001
**Orange Complex**	-		
*Fusobacterium nucleatum (Fn)*	87 72-96	84 69-94	Not significant
*Prevotella intermedia (Pi)*	66 49-80	37 22-54	0.001
